# Peculiar Deformity in a Horse, Due to Localized Muscular Atrophy

**Published:** 1880-01

**Authors:** James Hamill

**Affiliations:** 416 East Fourteenth Street, New York


### ART. II.—PECULIAR DEFORMITY IN A HORSE, DUE TO LOCALIZED MUSCULAR ATROPHY.

BY JAMES HAMILL, D. V. S.

MY first knowledge of the subjoined remarkable case was of a hearsay character. A friend last June related what seemed to him an unusual change in a horse, which the veterinary surgeon in attendance pronounced subcutaneous emphysema, and had failed to relieve. On my off-hand suggestion, that if the case was one of air in the subcutaneous cellular tissue, the treatment was very simple, and that punctures should be made, my informant told me that the latter procedure had been already resorted to, but that the animal, instead of improving, had steadily become worse, and was becoming more and more distended from day to day.

At his suggestion, I was called to see the case. On first sight, a layman would have been excusable if he had doubted that the animal in question was in reality a horse! Of tens of thousands of healthy and diseased horses that it has been my fortune to see even before adopting the veterinary profession, not one remotely exhibited anything like that found in this creature.

The upper (cartilaginous) border of the scapula *protruded fully eight inches above the withers*, when the weight was thrown on the fore-limbs. On the left side this border of the scapula was in place, but, owing to atrophy of the pectoral muscles and the great serratus, the scapulo-humeral articulation had moved about ten inches outwards from the normal topographical position. Let the reader imagine a horse, with the anterior half of its body sunk down on and between the anterior extremities, causing the left scapulo-humeral angle, which normally lies in an antero-posterior plane only, to lie at the same time in an angle, open inwards, and of about 110 degrees. Let him further imagine that on the right side the thorax has glided down on the inner face of the corresponding scapula, so that the latter, protruding, can be grasped through the skin as distinctly as if the limb were separated from the body, it appearing as if the sharp border of the bone must inevitably cut through the tense fold of skin which it has driven up before itself, and the reader will have an approximate idea of the shape of the animal that I first saw on the 20th of last June.

It could move about only with difficulty. When in motion, the body lurched from side to side, like a boat in a stormy sea. During action, the right or off scapula moved about loosely in every direction, apparently limited in its movements solely by the skin. The peculiar change in the left scapulo-humeral angle permitted the body to twist, as it were, on itself.

I obtained but a very imperfect history of the case. It seems that a slight swelling was first noticed between the fore-legs, in the pectoral region. The veterinarian called in, and who treated the case for two weeks before I saw it, pronounced the swelling to be emphysematous, or composed of air, as I stated. During his treatment it had continued to increase, and on taking charge myself, I found the swelling to be clearly oedematous, and extending from the shoulder and pectoral regions, along the inferior abdominal walls to and involving the sheath.* The day after I took charge the veterinarian previously in attendance arrived with two colleagues,† in my absence, and after holding a prolonged consultation, decided to have the horse shot, *in order to find out what was the matter with it.* The owner sent the animal to my infirmary that same day, walking him over a mile on a hot day. I on the *then* examination found the following: Temperature 103°, Respiration 60°, Pulse 71° per minute; breathing very labored, and as if impeded by some obstruction in the nasal passages. Owing to the enormous swelling an accurate examination was impossible, and I reserved my diagnosis, beyond the very *demonstrable* statement that the condition of the subcutaneous tissues was unquestionably one of oedema. It was, of course, out of question to attempt diagnosticating the condition of the thoracic viscera.

---

* As far as the diagnosis of my predecessor was concerned, I believe that I need not have hesitated to term it malpractice.

† One a "professor" in a veterinary school of this city.

He was much exhausted, doubtless owing to the owner's carelessness in permitting the amimal to walk the distance mentioned. I had the animal placed in slings, to rest the weakened scapular connections, after vainly endeavoring to devise some mechanical arrangement for the support of the displaced parts; without proper harness this had to be postponed. Such appliance would have been in the way of the topical treatment I employed.

I treated the case on general principles, first administering a powerful hydragogue cathartic, followed by large doses of iodide of potash. Locally I employed frictions, with absorbent remedies, over the entire area swollen.

On the 24th, the Respiration 33°, Pulse 54°, Temperature 103°; animal dull; does not feed at all; local treatment continued.

On the 25th (3d day): Temperature, 102½°; Respiration, 33°; Pulse, 54°; animal purging freely; swelling perceptibly diminished; obstruction in nasal passages much marked; these were syringed out with a mild solution of carbolic acid.

26th: Temperature, 102°; Respiration, 48°; Pulse, 60°; still refuses food; hypercatharsis, watery stools every few minutes; swelling rapidly decreasing. Did not attempt to check the purging.

27th: Temperature, 101°; Respiration, 53°; Pulse, 44°; general condition of animal appears better; purging ceases; remaining tumefaction *firmer* than before.

28th: Temperature normal; Respiration ditto; Pulse normal in frequency, but feeble. I began administering tonics, and introduced two setons in, and applied blisters over, the slight remaining swelling.

29th: Found animal a little feverish and restive; considerable discharge from the setons and blisters. This continued for three days, when, from the combined effect of the chafing slings and blisters, the lower aspect of the trunk became quite sore. I therefore removed the slings, and on walking the animal found motion improved. Treatment was now limited to the blistered or ulcerated surfaces, and attention to the general nutrition. The swelling entirely disappeared within a period of fifteen days from the time I commenced treatment. I did not re-apply the slings on account of the warm weather, but left the case to nature. My original prognosis had been, that while the animal would recover from the oedema, that on account of the already established atrophy of certain important muscles, it never would do for any severe work.

At present the deformity, although still visible, is nothing compared to what it originally was. The atrophied muscles have regained power to a great extent, and, far exceeding my most sanguine expectations, *I find the animal at the present date capable of doing light work.*

I have left the question of the essential pathological condition an open one. The suggestions of those whom I have conversed with on the subject are all more or less different among themselves. One considered it a complicated case of purpura; another, as a central nervous affection; a third, as the result of overwork. I can merely say, that not having seen the case early enough, nor having any data in reference to the early history, I reserve all conjecture. The one positive feature that I was interested in demonstrating, was that the swelling was *oedema*, not *emphysema.* Whether a proper treatment, applied on even general principles early enough in the disease, might not have led to a rapid and complete cure, I leave to the reader.

That the muscular atrophy was not spinal in its origin, is, I think, demonstrated by the recovery of certain of the weakened muscles in power. The left and right trapezius are, however, still entirely atrophic.

The question of the muscular mechanism of the deformity is a very interesting one. For the present I would merely remark, that the difference in the deformities of the two sides is to be very naturally explained by the different degree to which the symmetrical muscles were involved.

Thus, the tilting out of the left humero-scapular angle is referable to a greater atrophy of the pectorals on this side, perhaps also of the lower part of the *Mastoido Humeralis.* The gliding downwards of the thorax on the inner face of the right scapula is undoubtedly due to the atrophy of the great *Serratus*, which, as is well known, constitutes the powerful natural sling that holds the thorax on the anterior limbs in a normal state.

The case, therefore, assumes a considerable anatomical and physiological interest. The horse has passed into my possession, and is utilized for demonstrations in the College. At some later day I may be enabled to furnish a more elaborate essay on the case, accompanied by illustrative diagrams.

416 EAST FOURTEENTH STREET, NEW YORK.

